# Efficacy of Midazolam in Outpatient Pediatric Dentistry: A Systematic Review

**DOI:** 10.1111/scd.70107

**Published:** 2025-10-21

**Authors:** Judit Rabassa‐Blanco, Pau Cahuana‐Bartra, Yndira González‐Chópite, Maria Dolores Rocha‐Eiroa, Albert Ramírez‐Rámiz, Elias Isaack Mashala, Lluís Brunet‐Llobet, Jaume Miranda‐Rius

**Affiliations:** ^1^ Department of Dentistry Hospital Sant Joan de Déu University of Barcelona Barcelona Spain; ^2^ Department of Odontostomatology Faculty of Medicine and Health Sciences University of Barcelona Barcelona Spain; ^3^ Hospital Dentistry and Periodontal Medicine Research Group Institut de Recerca Sant Joan de Déu (IRSJD) Barcelona Spain; ^4^ Doctoral Programme in Medicine and Translational Research Faculty of Medicine and Health Sciences Hospital Sant Joan de Déu University of Barcelona Barcelona Spain

**Keywords:** conscious sedation, dental anxiety, midazolam, pediatric dentistry

## Abstract

**Aims:**

The aim of this systematic review was to analyze the efficacy, advantages and adverse effects of midazolam in outpatient pediatric dentistry.

**Methods:**

This review was carried out in accordance with the PRISMA criteria. A systematic electronic search was conducted through MEDLINE/PubMed, Scopus, and the Cochrane Library databases up to September 2024. An advanced and reproducible search strategy was used to identify relevant studies. Articles were excluded if they focused solely on midazolam as a premedication for general anesthesia or elective surgery, involving patients with special diseases. Inclusion criteria required participants aged 0–16 years, patients with behavioral and/or cooperation disorders and undergoing simple dental restorative procedures under local anesthesia, such as fillings, pulp therapies, stainless steel crowns, or basic extractions. Patients with specific medical conditions, as well as those who were not monitored for vital signs during sedation, were excluded from the study. The risk of bias assessment was analyzed using the criteria set out in the Cochrane Handbook for Systematic Reviews of Interventions, version 5.1.0.

**Results:**

A total of 28 studies were included in the analysis, which were conducted across 11 countries and involved a total of 4374 children aged between 2 and 14 years. Most studies demonstrated a low risk of bias. Many of the participants were ASA I or II status and were assessed using behavioral scales, primarily the Frankl scale. Twelve adjunct drugs were combined with midazolam, and various administration routes were explored, including oral, intranasal, and buccal. Dosing protocols varied, as did fasting guidelines prior to sedation. Outcome measures included vital sign monitoring and behavioral assessments, most commonly via the Houpt and MOAA/S scales. Midazolam generally proved effective in reducing anxiety and improving cooperation, with reported benefits extending to future dental visits. Adverse effects were infrequently noted and typically mild, including nausea, vomiting, and paradoxical reactions.

**Conclusions:**

Midazolam has been shown to be an effective and safe agent for moderate sedation in pediatric dental procedures when administered orally at a dose range of 0.3–0.5 mg/kg. The evidence suggests that it reliably reduces anxiety and improves cooperation. Supervision, preferably by an anesthesiologist, is recommended when combined with other drugs to ensure patient safety.

## 1 Introduction

Dental anxiety (DA) is an emotional state that causes unpleasant sensations in patients when thinking about dental treatments and may generate psychophysiological changes. The anticipation of dental treatment can generate feelings of distress and fear [1].

The prevalence of DA in the child‐juvenile population ranges between 5% and 20% and the condition is associated with higher‐than‐average levels of caries and poor oral health [1]. In addition, children's anxiety about dental procedures may cause behavioral problems that interfere with the success of the treatment. To relieve this anxiety and control the behavior of this population, pharmacological sedation and analgesia are often used [2].

Moderate sedation (MS) is a pharmacologically induced state, which occurs with depression of consciousness during which the patient responds correctly to verbal commands, both alone and accompanied by a light tactile stimulus. No additional interventions are needed to maintain a patent airway. Spontaneous ventilation is adequate. Cardiovascular function is generally maintained and correct‐ ASA I and ASA II patients may be considered candidates for conscious sedation on an outpatient basis. ASA III and IV patients represent special problems that require individual consideration and are best treated in an inpatient setting (Table 1) [3]. In pediatric dentistry, MS is used when non‐pharmacological management methods are ineffective. In such cases, the administration of certain pharmacological agents can guide the behavior of children during dental treatment. Since its effects wear off quickly, this type of sedation is used for simple minor surgery—that is, short‐duration surgical procedures generally applied to superficial structures which require the application of local anesthesia and entail low risk and minimal complications. MS is often preferred to general anesthesia due to its lower cost and risk, but its effectiveness depends on the medication and the method of administration [4]. A range of administration routes may be indicated, such as oral, intranasal, intravenous and inhalation, each with its advantages and disadvantages [5]. The induction of MS involves the use of anxiolytic drugs such as midazolam, ketamine, fentanyl, or dexmedetomidine, as a rule administered intranasally or orally [6, 7]. Midazolam is a benzodiazepine with anxiolytic, sedative, hypnotic, central muscle‐relaxant, and anterograde amnesic effects. It is considered an excellent sedative agent due to its favorable balance between toxicity and efficacy, the wide range of therapeutic doses, the wide safety margin, and a relatively short duration of action. These characteristics allow rapid induction of sedation and subsequent recovery in comparison with other agents [8]. However, as with other types of drugs that are taken orally, due to first‐pass hepatic metabolism, it is difficult to accurately calculate the effective absorbed dose; this makes the sedative effect difficult to adjust, with large individual differences and a highly variable success rate (ranging between 30% and 70%) [9]. The European Council of Dentists favors MS with nitrous oxide and oxygen as the recommended standard method of sedation [10], since onset and recovery are rapid, the dose can be adjusted, and the risks to body systems are minimal. However, successful administration of nitrous oxide depends on the practitioner's technical expertise and the patient's willingness to wear the mask, an issue that may cause difficulty in children with high levels of anxiety [11].

**TABLE 1:** ASA classification (American Society of Anesthesiologists).

ASA I	A normal healthy patient without physical or metabolic disorders.
ASA II	A patient with mild systemic disease that does not interfere with their daily activity.
ASA III	A patient with severe systemic disease not disabling and limiting daily activity.
ASA IV	A patient with severe systemic disease that is a constant threat to life.
ASA V	A moribund patient who is not expected to survive without the operation.

Midazolam may be associated with a variety of adverse effects. The most prominent are paradoxical reactions, nausea, vomiting, hiccups, oversedation, and mild respiratory depression depending on the dose administered [12, 13]. Though rare, these paradoxical reactions are characterized by emotional disinhibition, psychomotor agitation and in extreme cases aggressive behavior—the very opposite of the desired sedative effect [14].

An issue worth stressing is the cost saving offered by the possibility of treating certain pediatric patients with oral midazolam on an outpatient basis. This avoids the need for hospitalization and the use of an operating room and also makes general anesthesia unnecessary. Moderate sedation and general anesthesia are not direct substitutes for each other. General anesthesia is indicated when the volume of dental work exceeds what can be tolerated under sedation, or when the patient has medical conditions, anxiety or behavioral problems that preclude treatment under sedation.

The aim of this systematic review is to evaluate the efficacy of midazolam as a sedative in pediatric dental procedures and to provide comprehensive insights into its dosing protocols and potential adverse effects.

## 2 Materials and Methods

This review was carried out applying the PRISMA criteria (*Preferred Reporting Items for Systematic Reviews and Meta‐Analyses*), and the protocol was registered at the PROSPERO (CRD420245311747). Before conducting the literature search, we formulated the following PICO question: “Is midazolam effective in managing the behaviour of paediatric patients in a dental setting?” (Table 2).

**TABLE 2:** PICO question.

Population (P)	Intervention (I)	Comparison (C)	Outcome (O)
Child	Concious sedation with midazolam	Control. Placebo Other drugs Other methods	Better behavior in dentistry

**Search strategy**. An E‐search up until September 2024 was conducted using an advanced search: MEDLINE/PubMed, Scopus, and the Cochrane Library databases. A specific combination of words was entered in order to make the search reproducible (Table 3).

**TABLE 3:** Strategy for searching electronic literature.

Database	Keywords	Database filters	Results
MEDLINE Pubmed	((“Midazolam” [Mesh]) AND “Pediatric dentistry” [Mesh]) NOT “Review”	Article title, Abstract, Keywords	13
	((“Midazolam”) AND (Pediatric Dentistry)) NOT (Review)		199
	((Midazolam [Title/Abstract]) AND (Pediatric dentistry [Title/Abstract])) NOT (Review)		48
			260
Scopus	Midazolam AND “Pediatric dentistry” AND NOT Review	Article title, Abstract, Keywords	72
Cochrane	“Midazolam” (in Title Abstract Keyword) AND “Pediatric dentistry” (in Title Abstract Keyword)	Trials	82

**Type of studies**. We excluded papers whose titles or abstracts showed information not relevant to the research question: that is to say, studies on the exclusive use of midazolam as premedication prior to general anesthesia or elective surgeries; those of patients with special diseases, and those evaluating the drug's effect on emergence agitation. We also left out studies that were not accessible in full text using the SIRE tool. Finally, we excluded randomized clinical trials that had a sample size of 10 patients or fewer, in vitro and animal studies, systematic reviews, simple descriptions of techniques or protocols, editorials, opinions, reviews, case series, articles without a sample provided, incomplete clinical trials, and pilot studies. The risk of bias assessment was analyzed using the criteria set out in the Cochrane Handbook for Systematic Reviews of Interventions, version 5.1.0.

**Type of participants**. For a study to be included in this review, participants had to meet both of the following criteria: (i) patients aged 0–16 years; (ii) patients with behavioral and/or cooperation disorders; (iii) patients undergoing simple restorative treatments with local anesthesia (e.g., fillings, pulp treatments, stainless steel crowns) and/or simple extractions. Patients with specific medical conditions, as well as those who were not monitored for vital signs during sedation, were excluded from the study.

### 2.1 Type of Interventions

**Trial group**. Any type of sedative agent using the oral, buccal, inhalation, intranasal, sublingual, or rectal route of administration that can be applied by a dentist or anesthetist in a dental clinic or hospital setting. Studies reporting the use of deep sedation were excluded.

**Control group**. Placebo (no intervention), an alternative sedative agent, or a different dose of the same drug.

### 2.2 Outcome Measures

**Primary outcome measures**. Behavior is measured using different indices and scales. The Frankl scale was used in most cases, and on occasion, the Venham Test (VPT) and the Children's Fear Survey Schedule‐Dental Subscale (CFSS‐DS).

**Secondary outcome measures**. We assessed whether or not the treatment could be completed, the levels of sedation, postoperative anxiety, and adverse effects.

## 3 Results

### 3.1 Search Results

**FIGURE 1:**
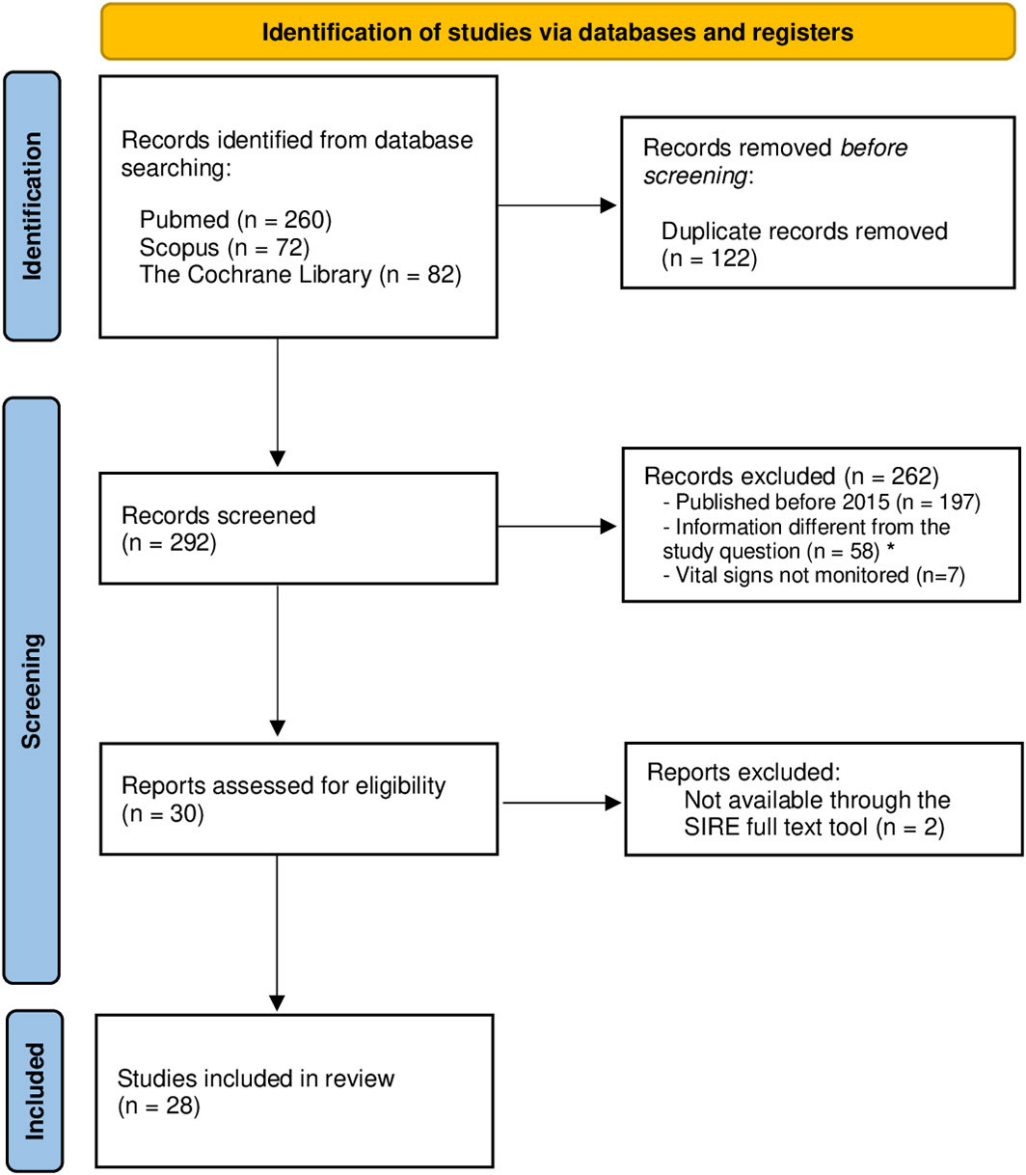
Flow chart of the study selection process (PRISMA).

Figure 1 presents the flow chart for the study selection process, following the PRISMA protocol. Initially, the results of the search databases were downloaded in RIS format (compatible with EndNote) to be imported into the bibliographic manager Zotero. In total, 414 articles were obtained. Subsequently, the “Duplicate items” tool was used to eliminate these repeated records, and a manual check was also carried out, resulting in the obtaining of 292 articles. The studies that did not address the research question posed or were not available in full text through the SIRE tool were excluded; applying this strategy, a total of 28 full articles were selected for further analysis.

### 3.2 Characteristics of the Studies

**FIGURE 2:**
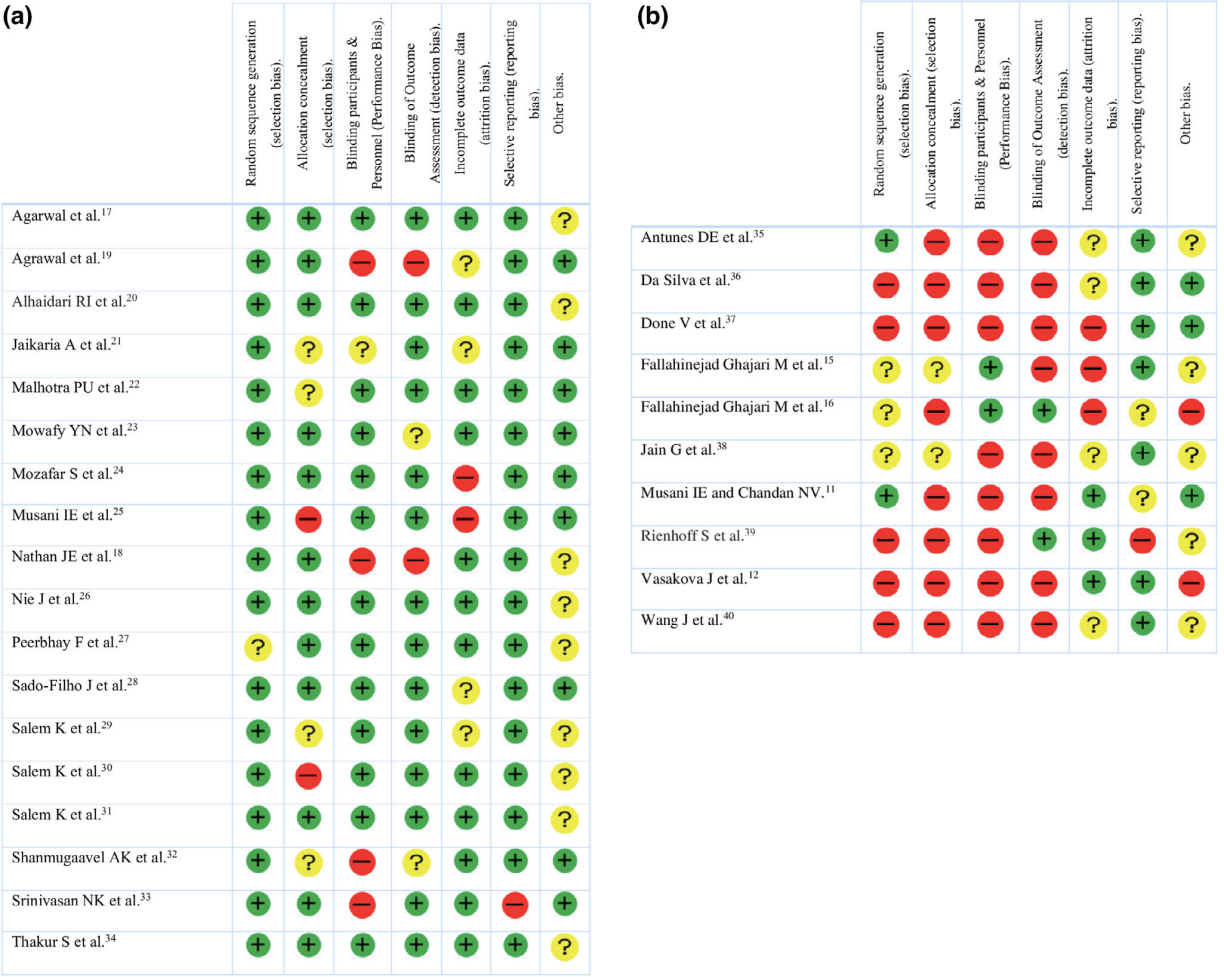
(a) Risk of bias assessment. According to the Cochrane Handbook for Systematic Reviews of Interventions, version 5.1.0. (+): low risk of bias; (–): high risk of bias; (?): uncertain risk of bias. Studies with low risk of bias (in alphabetical order). (b) Risk of bias assessment. According to the Cochrane Handbook for Systematic Reviews of Interventions, version 5.1.0. (+): low risk of bias; (–): high risk of bias; (?): uncertain risk of bias. Studies with high or unclear risk of bias (in alphabetical order).

The studies were carried out in 11 different countries, the largest proportion being performed in India (*n* = 10) and Iran (*n* = 6). Just over half the studies were randomized controlled trials (RCTs) (*n* = 17). The sample size of the different RCTs ranged from 16 participants [15, 16] to 128 participants [17]. The largest number of participants was recorded in the retrospective study by Nathan et al., which included 1785 patients [18] (Tables 4 and 5). The risk of bias was low in most studies (*n* = 18), high in 8, and unclear in two (Figure 2a,b).

**TABLE 4:** Studies with low risk of bias (in alphabetical order).

Author	Study	Objectives Scale used	*N*; sex; age; weight	Study groups; intervention	Conclusions
Agarwal et al. [17]	RCT DB	‐ Efficacy and safety by INS route: *MDZ‐K (MK)—*DEX‐K (DK) *MDZ‐F (MF)—*DEX‐ F (DF) ‐ Assessment of onset/depth of sedation, analgesia, conduct, treatment and RT. ‐Scale(s) used: UMSS/MOAA/S	‐ 128 (*n* = 32 in each group (MK, DK, MF, and DF). ‐ 4 to 9 years	• **4 groups**: ‐ 1: MK/INS at 0.2 mg/kg and 4.0 mg/kg. ‐ 2: DK/INS at 1 µg/kg and 1 mg/ ‐ 3: MF/INS at 0.2 mg/kg and 2 µg/kg ‐ 4: DF by INS route at 1 µg/kg (<100 µg) of DEX and 1.5 µg/kg (<100 µg) of F.	‐ DK and DF had greater analgesic‐sedative potential, faster onset and depth of sedation, and better intra‐ and post‐operative analgesia compared to MK and MF. ‐ DF had the fastest onset of sedation.
Agrawal et al. [19]	RCT	‐ Assessment of the anxiolytic efficacy of oral MDZ and a homeopathic remedy (*Aconite napellus*) during DT. ‐ Comparison of the ability of MDZ and the homeopathic remedy to reduce salivary cortisol and amylase levels, which are markers of anxiety.	‐ 48 (*n* = 24 in each group) ‐ 4 to 14 years	• **2 groups**: −1: Oral MDZ at 0.5 mg/kg, 20' before DT. −2: *Aconite napellus* at a potency of 30c, administered 60 min before DT.	− Both oral MDZ and the homeopathic remedy *aconite napellus* were effective in reducing anxiety during DT, as evidenced by decreased salivary cortisol, salivary amylase, HR and BP compared to baseline. *Aconite napellus* more effective than oral MDZ (NS). − *Aconite napellus* more palatable, easier to administer and safer with no known AEs.
Alhaidari et al. [20]	RCT DB C	− Assessment of the efficacy of F INS with oral MDZ in CS. − Behavioral scores with MDZ‐F versus MDZ alone. − Time to onset of sedation, working of sedation, behavior, and occurrence of AEs.—Scale(s) used: MOAA/S.	− 32 (18/14) − 54.6 months ±10.2 − 17.6 Kg ± 3.09	Assessment of sedation status and behavior when using the combination of F INS with oral MDZ versus oral MDZ alone. − Comparison of sedation onset time, working time and occurrence of AEs between the two sedation regimens.	− Oral MDZ and F‐INS sedation improved sedation and behavior during local anesthesia and DT compared to oral MDZ alone. − F‐INS prolonged the working time of sedation. − F‐INS safety: rapid onset, incorrect dose calculation, misuse comparable to parenteral route. − Use of F‐INS sedation should be carried out under strict and controlled clinical conditions.
Jaikaria et al. [21]	PS R TB	− Assessment and comparison of the sedative effect of oral combinations of MK, DEX‐F and DEX‐K in UC requiring DT. − Scale(s) used: MOAA/S.	− 34 − 3–9 years (4.59 ± 1.20 years) − 15.26 ± 2.41 kg	• **3 groups**: −1: *MK*: 0.3 mg/kg MDZ, 5 mg/kg K and honey. −2: *DF*: 2 µg/kg DEX, 3 µg/kg F and honey. −3: ‐*DK*: 2 µg/kg DEX, 5 mg/kg K and honey.	− MK, DF, and DK produced comparable sedation and behavior. − DK had a sedative property comparable to MK. − DF is a potential sedative agent in pediatric dentistry.
Malhotra et al. [22]	PS R DB	− Assessment and comparison of the effect of DEX INS and the combination of MK OR for CS in UC undergoing DT. − Combined with 30 mL mango juice. − Scale(s) used: MOAA/S.	− 36 − 3–9 years (4.60 ± 1.99 years) − 15.62 ± 4.21 kg	• **3 groups**: − 1: MK: 0.4 mL placebo INS. 0.5 mg/kg MDZ, 5 mg/kg K OR. − 2: DX: 1 µg/kg DEX INS. − 3: 0.4 mL INS saline solution.	− These were safe and effective combinations as sedative agents. − Hemodynamic parameters remained stable with both regimens. − GSSR, rate of satisfactory performance and rate of ease of completing DT were: MK > DEX > placebo.
Mowafy et al. [23]	RCT C	Assessment of the efficacy of aerosolized MDZ administered through the buccal mucosa versus INS mucosa in UC undergoing DT. − Scale(s) used: Wilton and Houpt.	− 36 (*n* = 18 in each group) − 3 to 5 years	• **2 groups**: −1: MDZ aerosolized buccal mucosa at a dose of 0.3 mg/kg. −2: INS aerosolized MDZ at 0.3 mg/kg. Alternating buccal/nasal for 1 week.	− Aerosolized buccal MDZ was better tolerated than INS MDZ. − INS route of MDZ: faster onset than oral route. − Both buccal and INS administration of aerosolized MDZ were safe and effective in sedating and controlling behavior in UCs during minor DT.
Mozafar et al. [24]	RCT C DB	− Comparison of the safety and efficacy of MDZ/N_2_O and promethazine/N_2_O for DT in UC. − Scale(s) used: Houpt.	− 18 (9/9). − 3 to 6 years (49.16 ± 14.9 months) − 15.57 kg	• **2 groups**: T1: G1: 0.5 mg/kg MDZ syrup + N_2_O; G2: 1 mg/kg promethazine syrup and N_2_O. T2: G1: 1 mg/kg promethazine syrup and N2O. G2: 0.5 mg/kg MDZ syrup and N_2_O.	− MDZ/N_2_O and promethazine/N_2_O combinations showed efficient and acceptable sedation results. − MDZ provided deeper sedation and improved behavior in the early phase of DT compared to promethazine. − The overall behavior during the final phase of DT did not differ significantly between the combinations.
Musani et al. [25]	RCT DB C	− Comparison of INS premedication with MDZ vs. control with saline and N_2_O sedation. − Assessment of whether MDZ premedication increases sedation with N_2_O (mask). − Scale(s) used: Houpt.	− 30 with *n* = 15 in the MDZ group and *n* = 15 in the saline group. − 4 to 8 years	• **2 groups**: 1: Premedication INS MDZ 0.1 mg/kg. 2: Sedation with N_2_O, in which 100% oxygen was administered for 1–2 m, followed by a gradual increase in the N_2_O concentration to 30%–50%.	− INS premedication with MDZ significantly improved nasal mask compliance, cooperation, and reduced limb movement and crying in children compared to the normal saline control group. − Use of MDZ premedication was safe and effective, and no AEs were reported.
Nathan et al. [18]	RS	− Assessment of the efficacy, relative safety, and varying levels of anxiety and uncooperative behavior of MDZ with and without MEP. − MEP can prolong the action/working time of MDZ, without restraint.	−1.785 −30–84 months, mean 48 months.	• **3 groups**: − 1: MDZ OR at 0.3–0.5, 0.7, and 1.0 mg/kg. − 2: MEP OR at doses of 1.0 and 1.5 mg/kg with MDZ. − 3: MDZ+MEP previous doses.	− MEP + MDZ can improve the quality, predictability, and safety of CS in patients with different levels of anxiety. In addition, it can significantly prolong the duration of sedation compared to MDZ alone. − Effective and safe dosing: MDZ at 0.7–1.0 mg/kg + MEP 1.0–1.5 mg/kg, guided by the patient's level of anxiety.
Nie et al. [26]	RCT DB	− Comparison of the sedative efficacy of oral MDZ alone and that of DEX INS plus oral MDZ in children with dental anxiety undergoing DT. Scale(s): Ramsay, Frankl and Houpt.	− 83 (32/51) − 3 to 12 years (6.1 years) A: 23.0 ± 9.5 B: 20.4 ± 6.7	• **2 groups**: − 1: DEX INS (2 µg/kg, max. 100 mcg) plus oral MDZ (0.5 mg/kg, max 20 mg) − 2: Oral MDZ (0.5 mg/kg, max 20 mg) plus normal saline INS.	− Oral MDZ (0.5 mg/kg)7DEX INS (2 µg/kg) had >GSSR vs. oral MDZ alone (0.5 mg/kg) in behavioral control. − The combination group had a slightly longer time to onset of sedation vs. the MDZ alone group. − No differences incidence of AEs between the two groups.
Peerbhay et al. [27]	RCT TB	− Comparison of the effectiveness and RT of 0.3 and 0.5 mg/kg of MDZ INS administered with a mucosal spray device. − Scale(s) used: Wilson.	− 118. − 4 to 6 years.	• **2 groups**: − 1: 0.5 mg/kg MDZ INS. − 2: 0.3 mg/kg MDZ INS.	− MDZ INS (0.3 or 0.5 mg/kg): safe and effective anxiolysis for emergency dental extractions in 4–6‐year‐olds. − The 0.5 mg/kg dose is more effective than the 0.3 mg/kg dose. − The 0.5 mg/kg dose had a RT > vs. 0.3 mg/kg. − 0.5 mg/kg dose should be used in hospital settings (ICU).
Sado‐Filho et al. [28]	RCT P TB	− Assessment of the efficacy of K INS (experimental) and MDZ OR as core elements for BC in preschool‐aged children during DT. − Scale(s) used: OSURBS.	− 84 (43/41) − 1 to 7 years (3.1 years) − 15.2 kg.	• **3 parallel groups**: − 1: KM INS: (4 mg/kg) and MDZ (0.2 mg/kg). (*n* = 26) − 2: *KMO* (control administration route): K (4 mg/kg) and MDZ (0.5 mg/kg). (*n* = 25) − 3: MDZ O: (1 mg/kg, max.20 mg). (*n* = 24)	Combination with MK appeared more effective than MDZ alone, for CS in UC during DT.
Salem et al. [29]	RCT DB	− Comparison of the behavioral and physiological effects of commercial MDZ syrup with an intravenous MDZ preparation administered OR in UC during DT. Scale(s) used: Houpt, NCBRS and CFSS‐D.	− 88 (41/47). − 4 to 7 years. − 20.35 ± 5.55 kg.	• **2 groups**: *< 5 years* (G1‐G2)—*> 5 years (G3‐G4)* − G1: 0.5 mg/kg MDZ sugar‐free syrup − G2: 0.5 mg/kg MDZ IV and orange syrup. − G3: 0.2 mg/kg MDZ sugar‐free syrup − G4: 0.2 mg/kg MDZ IV and orange syrup.	− Intravenous MDZ administered orally at doses of 0.2 to 0.5 mg/kg provided safe and effective sedation. − Lower doses (0.2 mg/kg) may be recommended for older children.
Salem et al. [30]	RCT P DB	Efficacy and safety of a commercial MDZ syrup with an IV or oral MDZ preparation. − Scale(s) used: Houpt, NCBRS and CFSS‐D.	− 88 (40/48) − 3 to 6 years. − A: 20.2 ± 5.4 kg B: 18.4 ± 3.9 kg	• **2 groups**: −1: >5 years: 0.2 mg/kg commercial MDZ syrup. −2: <5 years: 0.5 mg/kg intravenous preparation of MDZ administered OR.	− MDZ syrup and IV (OR) MDZ are safe and have desirable sedation effects. − IV (OR) MDZ at 0.2‐0.5 mg/kg provided safe and effective sedation in UC aged 3–6 years. − UC responded adequately to oral MDZ.
Salem et al. [31]	RCT DB P	− Assessment of the safety and efficacy of MDZ INS (atomizer) and DEX. − Evaluation of impact of dental fear and behavioral problems in sedation success using valid assessment tools.	− 92 G1 MDZ INS (*n* = 50) G2 DEX (*n* = 42) − 4–6 years	• **2 groups**: −1: DEX INS at a dose of 1 mcg/kg, with a maximum dose of 25 mcg (0.25 mL). 2: MDZ INS at a dose of 0.2 mg/kg, with a maximum dose of 5 mg (1 mL).	− MDZ INS performed more acceptably than DEX INS in patients with high dental fear. Psychological characteristics were not associated with sedation success. − Physiological parameters remained within normal limits in both the MDZ and DEX groups.
Shanmugaavel et al. [32]	RCT	− Assessment and comparison of changes in anxiety and acceptance after sedation with INS and sublingual MDZ. − Scale(s) used: Venham Test.	− 40 (24/16) − 3–7 years − A: 17.50 ± 4.39 kg B: 17.40 ± 4.33 kg	• **2 groups**: − 1: (*n* = 20): 0.2 mg/kg MDZ INS. − 2 (*n* = 2): 0.2 mg/kg MDZ sublingually.	− The sublingual route of administration was better accepted than the INS route. − During the administration of the local anesthetic, anxiety increased in both groups.
Srinivasan et al. [33]	RS C‐S	− Comparison of the efficacy of DEX INS with N_2_O vs. oral MDZ and oral MDZ combined with oral H with N_2_O. − Measurement of the effectiveness of sedation depth, duration, behavior, and procedures completed. − Scale(s) used: Ellis and Houpt.	− 146 (65/81) − 4.6 years (1.0) − 19.1 kg (3.9)	• **3 groups**: −1: DEX INS (3.0 mcg/kg) and N_2_O. −2: Oral MDZ (0.5–0.7 mg/kg) and N_2_O. −3: H oral (1.0 mg/kg, max. 25 mg), MDZ oral (0.5–0.7 mg/kg) and N_2_O.	− DEX INS with N_2_O showed no statistical difference in efficacy compared to oral MDZ or oral MDZ combined with oral H with N_2_O for CS in pediatric DT. − The majority of stimulating and prolonged procedures were completed with the DEX sedation regimen. − DEX had the longest onset time and working time of the sedation regimens.
Thakur et al. [34]	RCT DB EP	− Assessment of the efficacy of an oral combination of K and MDZ (1 mL of honey). − Assessment of the behavior and sedation scores of pediatric dental patients. − Scale(s) used: MOAA/S	− 36 (12 in each of the 3 groups) − 3–9 years (5.5 ± 1.30)	• **3 groups**: −1: 0.2 mg/kg of MDZ and 5 mg/kg of K (OR) −2: 0.3 mg/kg of MDZ and 3 mg/kg of K (OR) −3: 0.4 mg/kg of MDZ and 2 mg/kg of K (OR)	− The combination of 0.3 mg/kg MDZ and 3 mg/kg K (G2) had the highest GSSR (83.3%): − >91% of patients in all three groups showed better BC during DT with the MDZ and K combinations. − Ease of treatment completion was highest in G2, with 83.3%, followed by 66.7% in groups G1 and G3.

Abbreviations: AE, adverse effects/events; BC, behavior control; C‐S, cross‐sectional; C, crossed; CFSS‐DS, Dental Subscale of the Childrens’ CS, conscious sedation; CT, clinical trial; DB, double blind; DEX, dexmedetomidine; DT, dental treatment; ED, effective dose; ED_95_, dose needed to achieve the desired effect in 95% of the population; F, fentanyl; Fear Survey; GSSR, general sedation success rate; H, hydroxyzine; INS, intranasal; IV, intravenous; K, ketamine; MDZ, midazolam; MEP, meperidine; MH, midazolam/hydroxyzine; MK, midazolam/ketamine; MOAA/S, Modified Observer's Alertness/Sedation Scale; N_2_O, nitrous oxide; NCBRS, North Carolina Behavior Rating Scale; O_2_, oxygen; OR, oral route; OSURBS, Ohio State University Behavioral Rating Scale; P, parallel; PCS, prospective cohort study; PS, prospective study; R, randomized; RCT, randomized controlled trial; RS, retrospective study; RT, recovery time; SEM, sound, eye and motor scale; SpO_2_, oxygen saturation; TB, triple blind; TTS, total time of sedation; TUD, time until discharge; UC, uncooperative children; UMSS, University of Michigan Sedation Scale; VS, vital signs.

**TABLE 5:** Studies with high or unclear risk of bias (in alphabetical order).

Author	Study	Objectives Scale(s) used	*N*; sex; age; weight	Study groups. Intervention	Conclusions
Antunes et al. [35]	PS	− Comparison of the long‐term behavior of children undergoing different behavior techniques (non‐pharmacological control, moderate sedation or general anesthesia) during DT. − CS may improve cooperation in follow‐up by reducing distress during the initial DT. OSURBS scale.	− 50 (29/21) − <4 years. (2.8 mean) years at the beginning of the follow‐up period, and 4.3 years at the end of the same.	• **4 groups**: − 1: Without sedation (*n* = 17): control. − 2: OR: 1 mg/kg MDZ (<20 mg). (*n* = 16). − 3: OR: 0.5 mg/kg MDZ (<20 mg) and 3 mg/kg K (max. 50 mg). (*n* = 13). − 4: General anesthesia with sevoflurane, propofol, F. Premedication MDZ. (*n* = 4).	Patients undergoing CS with MDZ and K combination, showed more positive behavior during follow‐up dental visits compared to those who did not receive sedation. − The improved behavior in the sedated groups was probably due to the amnesic effects of the sedatives, which helped reduce the negative impact of the invasive DTs. − The group that received both MDZ and K showed the best behavior during follow‐up sessions.
Da Silva et al. [36]	CT P	− Assessment of the efficacy of CS vs. protective stabilization in UC. − OSUBRS scale: behavior, anxiety, pain and stress in the child, satisfaction/anxiety‐parents/dentist; and AEs. Cost‐effectiveness analysis.	−152 − 2 to <7 years, with subgroup analyses planned for 2–3 years and 4–6 years.	• **2 groups**: 1: CS with the oral combination of MDZ (0.5 mg/kg, <20 mg) and K (4.0 mg/kg, <100 mg). 2: Protective stabilization, in which the child's movements are restricted by an adult and a dental assistant during DT.	Considering the primary outcome, the hypothesis of this study is that sedated children have better behavior during DT than children whose behavior was controlled by protective stabilization without sedation.
Done et al. [37]	PCS	− Effectiveness of MDZ and oral K in combination with N_2_O—O_2_ in cooperative but apprehensive children undergoing DT. − Scale(s) used: Houpt.	− 30 − 3–9 years	• **2 visits**: · *first visit*: 0.5 mg/kg MDZ oral and N_2_O—O_2_. *· second visit*: 5 mg/kg K oral and N_2_O—O_2_.	− Oral MDZ/N_2_O and oral K/N_2_O are safe and effective. − No significant differences in HR, SpO_2_, RR. − The psychomotor effect of the MDZ group was slightly better than that of the K group.
Fallahinejad Ghajari et al. [15]	RCT DB	− Comparison of the efficacy of two doses of oral MDZ for CS in fearful children undergoing DT. − Scale(s) used: Houpt.	− 16 (6/10) − 3–6 years (48 months) − 16.2 kg	• **2 groups**: MDZ+1 mg/Kg H − 1: T1: 0.3 mg/kg MDZ syrup. T2: 0.5 mg/kg MDZ and 1 mg/kg H. − 2: T1 0.5 mg/kg MDZ T2: 0.3 mg/kg MDZ syrup 1 mg/kg H.	Oral GSSR was not significantly different in the 0.5 and 0.3 mg/kg MDZ/H combinations.
Fallahinejad Ghajari et al. [16]	RCT C DB	− Comparison of the efficacy and safety of the combination of MK INS and OR in combination with N_2_O for CS in children with high levels of dental anxiety. − Scale(s) used: Houpt	− 23 (18/5) − 3–6 years	• **2 groups**: − G1: T1: INS. 1 mL 2% lidocaine HCL, 0.25 mg/kg atropine, 0.5 mg/kg MDZ and 10 mg/kg K. T2: OR. 0.5 mg/kg MDZ, 10 mg/kg K and 0.25 mg/kg atropine. − G2: T1: OR. 0.5 mg/kg MDZ, 10 mg/kg K and 0.25 mg/kg atropine. T2: INS. 1 mL 2% lidocaine HCL, 0.25 mg/kg atropine, 0.5 mg/kg MDZ and 10 mg/kg K.	− The combination of MK INS produced a more satisfactory sedation for short DT (35 min) compared to OR. − The differences in the overall sedation levels were statistically significant at intervals of 15 and 30 min.
Jain et al. [38]	RS C‐S	Assessment of the effects of substituting MDZ for INS MDZ in the three‐drug combination of MEP, H, and MDZ (MEP‐H‐MDZ).	− 508 − 7.1 years G1 MEP‐H‐MDZ oral − Median age of 6.5 years for the MEP‐H‐MDZ INS group	• **4 groups**: − G1: Oral MEP‐H‐MDZ: simultaneous. − G2: MEP‐H: oral MEP and oral H. −G3: MEP‐H‐MDZ INS: MEP oral, H oral and MDZ INS 25–35'. − 4: MDZ only: oral MDZ or INS alone. Associated 35%–50% supplemental N_2_O.	− MEP‐H‐MDZ and MEP‐H‐MDZ INS more effective in sedating children than MDZ monotherapy. − Use of MDZ INS instead of oral MDZ in the three‐drug combination reduced time from medication administration to discharge by 23 min. − All sedation regimens: MEP‐H‐MDZ INS, elevated O_2_ saturation levels during DTs.
Musani and Chandan [11]	RCT C	− Comparison of two MDZ regimens (syrup/INS) with N_2_O—O_2_ to determine their efficacy, acceptability and safety in UC undergoing DT. − Scale(s) used: Ellis and Houpt.	− 30 (19/11) − 4–10 years	• **2 groups**: − G1: (*n* = 15): T1: 0.2 mg/kg syrup; T2: 0.1 mg/kg INS (0.5 mg MDZ / spray). − G2: (*n* = 15): T1: 0.1 mg/kg INS (0.5 mg MDZ/spray); T2: 0.2 mg/kg syrup (2 mg MDZ/1 mL syrup).	− MDZ OR syrup and MDZ INS spray were well accepted by children and easy to administer for dentists. − The INS route allowed for rapid onset of action and rapid patient recovery compared to the OR route. − MDZ administered intravenously was a good alternative to oral administration and was equally effective at lower doses.
Rienhoff et al. [39]	RS	− Assessment of whether MDZ + hypnosis could improve the behavior of anxious children in multiple DT sessions. − Scale(s) used: Venham and Wong‐Baker.	− 311 (169/142) − 3–12 years.	MDZ at a dose of 0.4 mg/kg combined with various hypnosis techniques: animal‐force induction, color induction, bird‐swinging induction, and magic arm induction.	− Combined hypnosis and CS were effective in reducing anxiety during DT. Low doses of MDZ and hypnosis were effective. − Children's self‐assessment of their well‐being did not match their observed behavior during treatments.
Vasakova et al. [12]	RS	− Description of the effects of MDZ on VS in children, the occurrence and incidence of AEs, the relationship between the intervention and behavior, and the possible relationship between the degree of impairment and behavior. − Scale(s) used: SEM.	− 272 (139/133) − 1–12 years (5.5 years) − 20.7 kg	0.5 mg/kg (max. 12 mg) MDZ OR.	− MDZ OR 0.5 mg/kg is safe, even in children younger than the study ages. − Decreases BP and HR within the physiological range. − Speed of action is age‐dependent. − The older the child, the better his/her behavior. − Unwillingness to receive MDZ is a predictor of uncooperativeness.
Wang et al. [40]	PCS	− Measurement of the ED_95_ of esketamine INS with 0.5 mg/kg oral MDZ. − Assessment of secondary outcomes including sedation onset time, treatment time, **awakening** time, and incidence of AEs. − Scale(s) used: MOAA/S.	− 60 (27/33) − 51.5 months (34.5–64.0) − IMC: 17 (16–18)	• **2 groups**: −1: MDZ oral solution (0.5 mg/kg). −2: Esketamine INS, with an ED_95_ dose determined at 1.99 mg/kg.	− The ED_95_ of INS esketamine with 0.5 mg/kg oral MDZ for CS was 1.99 mg/kg (95% CI: 1.95‐2.01 mg/kg). − T 1/2 of sedation onset: 43.7 ± 6.9 min, 15.0 (10‐24.0) min for examination and 89.4 ± 19.5 min for awakening. − The incidence of intraoperative nausea/vomiting was 8.3%. AEs such as transient hypertension and tachycardia occurred during interventions.

Abbreviations: AE, adverse effects/events; BC, behavior control; C‐S, cross‐sectional; C, crossed; CFSS‐DS, Dental Subscale of the Childrens’ CS, conscious sedation; CT, clinical trial; DB, double blind; DEX, dexmedetomidine; DT, dental treatment; ED, effective dose; ED_95_, dose needed to achieve the desired effect in 95% of the population; F, fentanyl; Fear Survey; GSSR, general sedation success rate; H, hydroxyzine; INS, intranasal; IV, intravenous; K, ketamine; MDZ, midazolam; MEP, meperidine; MH, midazolam/hydroxyzine; MK, midazolam/ketamine; MOAA/S, Modified Observer's Alertness/Sedation Scale; N_2_O, nitrous oxide; NCBRS, North Carolina Behaviour Rating Scale; O_2_, oxygen; OR, oral route; OSURBS, Ohio State University Behavioral Rating Scale; P, parallel; PCS, prospective cohort study; PS, prospective study; R, randomized; RCT, randomized controlled trial; RS, retrospective study; RT, recovery time; SEM, sound, eye and motor scale; SpO_2_, oxygen saturation; TB, triple blind; TTS, total time of sedation; TUD, time until discharge; UC, uncooperative children; UMSS, University of Michigan Sedation Scale; VS, vital signs.

### 3.3 Characteristics of the Participants

The mean number of participants per study was 156 (standard deviation (SD) = 323) with a total of 4374 children in the 28 studies included.

The age of the participants ranged from 2 to 14 years. The most frequent age ranges were 3–4 and 6–7 years (47%). The weight (average 16.9 kg) was recorded in 12 studies, but the body mass index was recorded in only one [40].

The ASA status of the participants was detailed in 25 studies, of which 12 included only patients classified as ASA I, while 13 included both ASA I and ASA II patients. Most studies used the Frankl scale (*n* = 15) to assess patients’ behavior. Of these, the majority (*n* = 13) were classified as negative (grade II) or definitely negative (grade I).

Three studies used other assessment tools, such as the Venham Test [27, 32] and the CFSS‐DS [31]. In 10 studies, the use of standardized scales was not specified, and the assessment was based on subjective observations of uncooperative behavior.

Four studies reported the use of protective stabilization (PS) to immobilize children during dental treatment [18, 20, 23, 35]. Two studies [32, 36] reported the combination of PS together with behavior management techniques. Twelve studies reported that they did not use PS, and 10 more studies did not use either PS or behavior management techniques.

### 3.4 Characteristics of the Interventions

Twelve other drugs were used in combination with midazolam: ketamine, meperidine, fentanyl, dexmedetomidine, N_2_O, atropine, hydroxyzine, promethazine, chloral hydrate, morphine and diazepam, and one homeopathic remedy (*aconite napellus*).

The medications were administered orally (*n* = 13), intranasally (*n* = 5), buccally (*n* = 1), orally plus intranasally (*n* = 8), and finally sublingually plus intranasally (*n* = 1). The studies evaluated the action of different doses of midazolam either alone or in combination with other drugs (*n* = 16), comparing different routes of administration (*n* = 3) and exclusive use at different doses (*n* = 7). One study evaluated the use of midazolam with hypnosis [39] and another compared midazolam with a homeopathic remedy [19].

In 16 studies (57.1%), a period of fasting (NPO) was performed prior to sedation. The duration of NPO was variable, but most proposed a 6‐h fast from solid food and a 2‐h fast from liquids (*n* = 12). Other authors used a fasting period of 6 h for solids and 4 h for liquids [16]. Musani et al. mentioned dietary guidelines before sedation, but did not specify anything else [25]. Vasakova et al. required maintenance of fasting for 2 h after sedation [12]. Peerbhay et al. did not require NPO [27]. The other 11 studies did not refer to NPO.

Most studies (*n* = 17) reported the types of dental treatment performed: pulpal (*n* = 4), extractions (*n* = 2), restorative (*n* = 3), pulpal treatments and reconstructions or crowns (*n* = 3) and finally, pulpal treatments and/or crowns and/or fillings and/or extractions (*n* = 5). The remaining studies (*n* = 11) did not specify the types of treatment performed.

Most of the conscious sedations in the studies were performed or supervised by the anesthetist (*n* = 14), while in others (*n* = 8) MS was performed by the dentists themselves. Only one study reported that the prior assessment of the patient had been carried out by an anesthetist [25]; the remaining studies (*n* = 9) did not specify whether the sedation was performed by a dentist or by an anesthetist.

### 3.5 Characteristics of Outcome Measures

Most studies (*n* = 26) referred to monitoring vital signs: heart rate (*n* = 24), respiratory rate (*n* = 9), oxygen saturation (*n* = 25), and blood pressure (*n* = 18).

Agrawal et al. also analyzed salivary cortisol and salivary alpha‐amylase in their study and Srinivisan et al. analyzed end‐tidal CO_2_ [19, 33].

A positive initial contact with dental treatment and midazolam‐induced amnesia encourages greater cooperativeness at subsequent visits. Antunes et al. found that 37% of patients who were given midazolam syrup at the first visit behaved “calmly” at a visit 10 months later, presenting a less negative response to invasive dental treatment [35].

A wide variety of scales were used to measure the main outcomes proposed (i.e., the degree of sedation, the degree of anxiety and cooperation/behavior during treatment). Many studies used the same scale to evaluate the degree of sedation and patient cooperation. The most frequently used was the Houpt scale (*n* = 12) followed by the Modified Observer's Assessment of Alertness/Sedation scale (MOAA/S) (*n* = 6). Only four studies did not record the scales used. Some studies also evaluated pain using the Face, Legs, Activity, Cry, Consolability (FLACC) scale [17, 36] and the Wong‐Baker FACES Pain Rating Scale [39].

Most studies assessed the efficacy of different doses or routes of administration of midazolam in terms of the time required to achieve sedation, the total duration, the degree, and the recovery time. Adverse effects were mentioned in 9 studies, the most common being postoperative nausea and vomiting, hallucinations, hiccups, and paradoxical reactions.

## 4 Discussion

In the 28 studies analyzed in this review, the design and reporting were mostly acceptable, although recurrent biases were found in selection, execution, and detection. The description of the participants was poor in several studies, which failed to report data such as weight and anxiety status prior to sedation. In contrast, most studies reported the ASA status and the patient's behavior prior to sedation. The description of participants was poor in several studies, which did not report data such as weight and anxiety status prior to sedation. In contrast, most studies reported ASA status and patient behavior prior to sedation. The latter is an indicator of the degree of cooperation.

Several studies did not refer to the fasting period, and not all followed the same protocol, although most performed fasting in accordance with the American Academy of Pediatric Dentistry (AAPD) guidelines [5]. The fasting period (NPO) is crucial to ensure the effectiveness and safety of sedation, especially when administered orally. The lack of prior fasting may alter the absorption and effects of the medication and may affect the results of sedation. Without adequate fasting time, food or liquids in the stomach can interfere with the pharmacokinetics of sedatives, affecting their action. Moreover, fasting significantly reduces the risk of vomiting and aspiration during or after sedation, which are major concerns due to their potential to cause serious airway complications. By minimizing the presence of stomach contents, fasting helps prevent respiratory issues and ensures a safer sedation experience overall. Therefore, compliance with fasting recommendations is essential to optimize the effects of sedation and to ensure a safe, controlled intervention.

The dental treatments performed varied considerably in terms of complexity. These differences may well have influenced the patient's behavior and level of anxiety, since inserting a filling is not the same as performing an extraction. Nevertheless, participants generally completed the treatment, regardless of the group to which they were assigned. Some studies reported the use of protective stabilization, which allows a reduction in the dose of the sedative drugs needed and, in certain cases, may even avoid the need for MS when treatment can be performed quickly. The implementation of these behavioral techniques with children may allow effective and minimally invasive management and entail a lower risk of adverse effects due to sedation.

Most of the studies included performed sedation in children aged from 3 to 7 years. The treatment and management needs of children vary as they grow, develop, and mature psychologically; techniques that are appropriate for a 3‐year‐old may not be appropriate for a 12‐year‐old and vice versa [29, 30]. Therefore, several studies proposed different doses depending on age.

In most cases, the children were healthy or had mild systemic disease (ASA I and II). More than half of the studies classified patients’ behavior as “negative” or “definitely negative” on the Frankl scale. This scale has four categories; categories 1 and 2 correspond to uncooperative patients, while categories 3 and 4 represent cooperative patients.

It would have been desirable to exclude crossover trials since the level of anxiety and initial behavior in the second phase of treatment depends largely on the success or failure of the first period. The interpretation of the data on behavioral outcomes was difficult because, although half of the studies used the Houpt scale to record the degree of sedation/behavior, the others used a wide range of different instruments. In addition, behavior was recorded throughout treatment in some studies but only at specific times in others. The statistical methods used also varied, although the results were often similar.

Overall, the studies included in this review support the efficacy of the use of midazolam in achieving moderate sedation in uncooperative children and in improving their behavior in the consultation room.

### 4.1 Midazolam Alone

#### 4.1.1 Oral Administration

As a rule, midazolam was administered orally, in a range of preparations such as syrups and sprays (*n* = 19).

After administration of midazolam in syrup, 32.1% of patients demonstrated “calm behavior” [15]. Behavior was “very good” in between 43.2% and 70% [12, 25, 33] and “excellent” in 29.5% [12]. Regarding vital signs, Vasakova et al. reported that the administration of midazolam reduced blood pressure and increased heart rate, though the differences were not clinically significant [12]. Salem et al. and Sado‐Filho et al. observed that the physiological parameters after administration of midazolam were within the normal range [28, 29, 30]. Nevertheless, **oral suspension** is not a viable option in many countries, and as a result, intravenous preparations are administered orally. Salem et al. concluded that this preparation in doses of 0.2–0.5 mg/kg was a safe and effective regimen that achieved a reasonable level of sedation; however, the palatability was lower (59%) than that of commercial syrup (79.5%) [30]. Oral administration of midazolam in the form of a **spray** was reported in three articles, in which the authors described spraying different areas and doses (0.3 mg/kg in the buccal vestibules or 0.2 mg/kg under the tongue) and suggested that it can be safely used for sedation in children. Shanmugaavel et al. reported scores of zero on the Venham scale after administration [32].

#### 4.1.2 Intranasal Administration

Five studies analyzed the intranasal administration of midazolam [25, 27, 32]. Peerbhay et al. observed that intranasal administration of 0.5 mg/kg of midazolam achieved greater anxiolysis than a dose of 0.3 mg/kg. The 0.5 mg/kg group presented lower scores on the behavior scale at the time of administration of the local anesthetic and at the time of extraction. The authors concluded that the 0.5 mg/kg dose was more effective than the 0.3 mg/kg dose in reducing anxiety and improving the child's behavior [27]. Other studies indicated only that the administration of 0.2 mg/kg of intranasal midazolam produced acceptable sedation, without determining the type of treatment performed [30, 32]. Regarding the acceptability of the intranasal application, Peerbhay et al. observed that 35% of patients were relaxed and happy, a figure that rose slightly to 40% in the study by Shanmugaavel et al. [27, 32]. The most prevalent side effect was nasal discomfort, with a burning sensation present in 38.2% of patients [27] and coughing or sneezing in 16% [27].

### 4.2 Midazolam in Combination

The combination of midazolam and other drugs aims to benefit from the synergistic effects to achieve optimal sedation and cooperation.

Theoretically, the addition of nitrous oxide (N_2_O) allows the administration of lower doses of midazolam to achieve the same result. Five studies analyzed midazolam in combination with N_2_O and oxygen [11, 24, 33, 37]. In all cases, the dose of midazolam administered was 0.5 mg/kg, except in the study by Musani et al., who obtained “very good” to “excellent” rates of cooperation at a lower dose (0.2 mg/kg). A concentration of 50% N_2_O and 50% O_2_ provides an anxiolytic and analgesic effect. Musani and Chandan concluded that the midazolam and N_2_O regimen is safe and effective, greatly reduces patient anxiety, and improves the behavior of uncooperative children [11].

Regimens combining the anxiolytic effect of midazolam and the analgesic property of ketamine aim to achieve lower levels of anxiety and better behavior than when using these drugs alone [16, 17, 35, 36, 22, 28, 34]. Sado‐Filho et al. found that 50% of children behaved calmly for at least 60% of the treatment, considering sedation successful and superior to that achieved with midazolam alone (32.1%) [28]. Agarwal et al. showed that the combination of midazolam and ketamine had a greater analgesic‐sedative potential, faster onset, and greater depth of sedation than other drug combinations [17]. In the oral administration of this combination, the studies used doses of 0.5 mg/kg of midazolam, except for Thakur et al., who administered a dose of 0.3 mg/kg and obtained a higher overall success rate [34]. Between 83.3% and 90.9% of children presented improved behavior during treatment [21, 22]. Antunes et al. reported “calm behavior” in 50% of children 10 months after the first visit, a figure higher than that achieved with the administration of midazolam alone. Thus, the combination of ketamine with midazolam seems to be more effective in guiding the behavior of children during dental treatment than midazolam alone [35].

Some authors report the use of combinations of midazolam with other drugs: hydroxyzine, meperidine, dexmedetomidine, fentanyl, and others [26]. Combining midazolam with an antihistamine such as hydroxyzine or promethazine may reduce the risk of postoperative nausea and vomiting as a consequence of the sedation, and its combination with meperidine significantly prolongs MS and may improve its quality, predictability, and safety.

In this review, the majority of sedations were administered and monitored by an anesthetist, particularly due to the frequent use of drug combinations that require specialized knowledge to manage potential interactions and adverse effects. In many countries—excluding, for example, the United States—dentists are not authorized or trained to perform sedation involving multiple pharmacological agents. While the involvement of an anesthetist enhances safety, it may also increase the overall cost of care, potentially creating financial barriers for some families and limiting access to sedation‐supported dental treatments.

Nonetheless, increasing the interaction between the disciplines of medicine and dentistry in the hospital setting is key to improving the quality of treatment. This close collaboration not only optimizes the coordination of patient‐centered care, but also encourages the exchange of knowledge between disciplines, which enriches the technical skills of each professional. Regular interaction with other specialists in the hospital allows pediatric dentists to keep up to date with medical and dental advances, thus improving their ability to address complex cases and offering more comprehensive and effective care to their patients.

### 4.3 Ideal Dose of Midazolam in Pediatric Dentistry

The dose of midazolam should be individualized, considering not just the patient's weight but also their age and degree of anxiety and the desired level of sedation [38]. With age, as communication skills improve, so does behavior; it may be desirable to lower the dose so as to reduce the risk of adverse effects, and to reserve higher doses for patients who do not respond favorably to lower doses. In general, the recommended oral dose of midazolam is in the range of 0.2–1 mg/kg. The most commonly used oral dose is 0.5 mg/kg with a maximum dose of 20 mg. At this concentration, side effects are minimal and sedation satisfactory, with higher average success rates than those achieved with lower doses [12, 20]. Furthermore, intranasal administration achieves a faster onset of action and recovery than oral administration [16, 25, 38 ].

Thirteen of the studies included in this review reported adverse events, though not in a uniform manner. The most common adverse effect was nausea and vomiting, observed in 25.7% of the studies [16, 17, 21, 22, 28, 40] and less frequent postoperative hallucinations [22, 28], malaise [21], drowsiness [16, 28], paradoxical reactions [12, 28], irritability [28], and hiccups [33] were also recorded.

In this review, several limitations were identified in the studies analyzed. One of the most significant issues was the inconsistency in the reporting of participant data, such as weight and anxiety status prior to sedation, which was often omitted, despite the importance of these factors in determining the effectiveness of sedation. Additionally, although most studies adhered to fasting guidelines as per the American Academy of Pediatric Dentistry (AAPD), the lack of standardization in fasting protocols across studies was noted as a potential confounder that could affect the pharmacokinetics of sedatives (MS) and their subsequent effectiveness. The variability in the complexity of dental treatments performed across studies, ranging from fillings to extractions, also made it challenging to assess the true impact of sedation on patient behavior, as the level of anxiety and cooperation could differ based on the procedure. Another limitation was the inconsistent use of behavior scales to assess sedation outcomes, with some studies utilizing the Frankl scale, while others used different tools, making comparisons difficult. Furthermore, the presence of crossover trials in some studies introduced biases in interpreting behavioral outcomes, as the results could be influenced by the success or failure of the initial treatment phase. Finally, although midazolam was found to be effective in promoting sedation and cooperation, the variations in dosage, administration routes, and the use of additional medications complicate the ability to draw definitive conclusions regarding the ideal sedation regimen.

## 5 Conclusions

This systematic review highlights the effectiveness of midazolam as a reliable agent for moderate sedation in pediatric dental procedures. When administered orally at doses ranging from 0.3 to 0.5 mg/kg, midazolam demonstrates a wide safety margin and consistent clinical efficacy in reducing anxiety and enhancing cooperation. Its use, particularly in combination with other pharmacological agents, should be carefully supervised, preferably by an anesthesiologist, to minimize the risk of drug interactions and adverse effects, thereby ensuring optimal patient safety and treatment outcomes.

## Author Contributions

J.R.‐B., P.C.‐B., M.D.R.‐E., and A.R.‐R. conducted an extensive literature search and meticulously selected the manuscripts for review. Drafting of the article was undertaken collaboratively by J.R.‐B., P.C.‐B., Y.G.‐C., M.D.R.‐E., A.R.‐R., E.I.M., L.B.‐L., and J.M.‐R. Substantive manuscript revisions were carried out by J.R.‐B., L.B.‐L., and J.M.‐R. The final manuscript was reviewed and approved by all authors.

## Ethics Statement

No ethical approval or consent to participate was deemed necessary for this manuscript.

## Consent

Obtaining consent for publication was not a requisite for this manuscript.

## Conflicts of Interest

The authors declare no conflicts of interest.

## Data Availability

For those interested, the data sets used and/or analyzed during the present study can be obtained by contacting the first or corresponding author.
